# A Cost Effectiveness Analysis of Salt Reduction Policies to Reduce Coronary Heart Disease in Four Eastern Mediterranean Countries

**DOI:** 10.1371/journal.pone.0084445

**Published:** 2014-01-07

**Authors:** Helen Mason, Azza Shoaibi, Rula Ghandour, Martin O'Flaherty, Simon Capewell, Rana Khatib, Samer Jabr, Belgin Unal, Kaan Sözmen, Chokri Arfa, Wafa Aissi, Habiba Ben Romdhane, Fouad Fouad, Radwan Al-Ali, Abdullatif Husseini, the MedCHAMPS project team

**Affiliations:** 1 Yunus Centre for Social Business and Health, Glasgow Caledonian University, Glasgow, United Kingdom; 2 Institute of Community and Public Health, Birzeit University, Birzeit, Palestine, Occupied Palestinian territory; 3 Department of Public Health and Policy, Institute of Psychology, Health and Society, University of Liverpool, Liverpool, United Kingdom; 4 Department of Health Economics, Ministry of Health, Nablus, Palestine, Occupied Palestinian territory; 5 Dokuz Eylül University Faculty of Medicine, Department of Public Health, İnciraltı- İzmir, Turkiye; 6 Narlidere Community Health Center, Provincial Health Directorate of Izmir, Izmir, Turkey; 7 INTES/University of Carthage, Tunis, Tunisia; 8 Cardiovascular Disease Epidemiology and Prevention Research Laboratory, Faculty of Medicine, University Tunis El Manar, Tunis, Tunisia; 9 Syrian Center for Tobacco Studies, Aleppo, Syria; 10 Public Health Program, Department of Health Sciences, Qatar University, Doha, Qatar; Groningen Research Institute of Pharmacy, Netherlands

## Abstract

**Background:**

Coronary Heart Disease (CHD) is rising in middle income countries. Population based strategies to reduce specific CHD risk factors have an important role to play in reducing overall CHD mortality. Reducing dietary salt consumption is a potentially cost-effective way to reduce CHD events. This paper presents an economic evaluation of population based salt reduction policies in Tunisia, Syria, Palestine and Turkey.

**Methods and Findings:**

Three policies to reduce dietary salt intake were evaluated: a health promotion campaign, labelling of food packaging and mandatory reformulation of salt content in processed food. These were evaluated separately and in combination. Estimates of the effectiveness of salt reduction on blood pressure were based on a literature review. The reduction in mortality was estimated using the IMPACT CHD model specific to that country. Cumulative population health effects were quantified as life years gained (LYG) over a 10 year time frame. The costs of each policy were estimated using evidence from comparable policies and expert opinion including public sector costs and costs to the food industry. Health care costs associated with CHDs were estimated using standardized unit costs. The total cost of implementing each policy was compared against the current baseline (no policy). All costs were calculated using 2010 PPP exchange rates. In all four countries most policies were cost saving compared with the baseline. The combination of all three policies (reducing salt consumption by 30%) resulted in estimated cost savings of $235,000,000 and 6455 LYG in Tunisia; $39,000,000 and 31674 LYG in Syria; $6,000,000 and 2682 LYG in Palestine and $1,3000,000,000 and 378439 LYG in Turkey.

**Conclusion:**

Decreasing dietary salt intake will reduce coronary heart disease deaths in the four countries. A comprehensive strategy of health education and food industry actions to label and reduce salt content would save both money and lives.

## Introduction

Coronary heart disease (CHD) is rapidly increasing in middle income countries, and by 2020 deaths are predicted to overtake those from infectious diseases in all regions except Sub-Saharan Africa [1]. This reflects an increase in major cardiovascular risk factors, particularly rising levels of blood pressure, cholesterol, obesity and diabetes as a consequence of changes in nutrition and decreases in physical activity, compounded by high tobacco use. The Eastern Mediterranean Region (EMR) has been recognised as a hot-spot for CHD, where disease projections will exceed those of other regions [1].

There is now a pressing need to develop population based policies to reduce the burden of CHD. This was highlighted by the UN High Level meeting on Non Communicable Diseases in September 2011 [2], in particular, focusing on the key modifiable risk factors including salt intake.

Diets high in salt increase blood pressure levels which is the leading contributor to cardiovascular disease mortality [3]. Decreasing dietary salt intake from 10 grams to 5 grams per day could reduce cardiovascular diseases rate by 17% worldwide [4].

Dietary salt can come from two main sources: in the preparation and serving of food at home, or through manufacturers adding it during the processing of the food products. Strategies to reduce dietary salt intake therefore require a combination of policies depending on which sources of salt consumption are most prevalent within the specific country.

There are a number of potential policies governments can introduce to reduce dietary salt intake. Health promotion campaigns and labelling of food packaging with the salt content can help to raise awareness and encourage individuals to reduce salt consumption. Governments can also work with manufacturers to encourage voluntary reformulation of processed food products or go further and set mandatory regulations on the amount of salt in processed food products. Fiscal policies like taxation of high salt products might also be considered [5], [6].

One of the most important considerations when deciding on what salt reduction policy to adopt is cost effectiveness. Increasing demands are being placed on health care systems around the world as a result of aging populations and advances in expensive technologies. Governments must therefore consider both the costs and the benefits of all policies implemented. Such considerations therefore become even more critical in low and middle income countries where resources are particularly scarce.

There is increasing evidence on the cost effectiveness of these salt reduction policies in high income countries [6], [7]. In Australia, one study concluded that reformulation of processed food either through voluntary agreement between government and the food industry or through mandatory regulations for industry was cost saving; dietary advice alone was apparently not cost effective [8]. A Norwegian modelling study suggested that a combination of policies including an information campaign, working with industry to reduce salt in food products and taxation of high salt products increased life expectancy and was cost saving [9]. Similarly a modelling study in the US suggested that government collaboration with manufacturers to reduce salt in processed food could gain two million additional quality adjusted life years and annually save over US$32 billion in medical costs [10].

However, much less research has been conducted in middle income countries. These populations are often at different stages within the epidemiological transition. Furthermore, their health care systems face different resource constraints reducing the applicability of the results from high income countries. Asaria et al [11] modelled the cost effectiveness of a voluntary reduction in salt content of processed food by manufacturers supported by a media campaign in 23 low and middle income countries. Assuming that this strategy would lead to a 15% reduction in salt intake, they estimated 8.5 million deaths would be averted across the 23 countries over 10 years at a cost of between US$0.04 and US$0.32 per person per year depending on the country. Murray et al [12] performed an analysis using selected WHO regions. They evaluated two policies; voluntary agreements with manufacturers and then mandatory legislation to reduce salt content in products. For the EMR B region [13] (the focus of this paper) resulted in an annual cost of PPP$54 per disability adjusted life year (DALY) averted for the voluntary policy and PPP$27 per DALY averted for the mandatory legislation. However both studies excluded health care costs from the analysis making it difficult to make direct comparisons between the results for low, middle and high income countries.

Given this paucity of evidence, this paper presents the first detailed study evaluating the cost effectiveness of a range of policies to reduce dietary salt intake in four middle income countries in the Eastern Mediterranean. Dietary salt intake in all four countries is high (approximately 14 g per day in Tunisia, Syria and Palestine and 18 g per day in Turkey). Therefore, there is potential to have a substantial impact on salt intake. Our evaluation used IMPACT, the most widely published CHD policy model [14], [15]. The IMPACT CHD model is a cell based deterministic model which is comprehensive and includes all patient groups, all standard treatments and all major risk factors. It can be used to explain past trends in CHD and generate predictions of future trends. Country specific IMPACT models have been developed as part of the MedCHAMPS (MEDiterranean studies of Cardiovascular disease and Hyperglycaemia: analytical Modelling of Population Socio-economic transitions) project [16]. These country specific models were used to evaluate the potential health benefits and the cost effectiveness of different salt reduction policies.

## Methods

### Potential policies for evaluation

A number of potential policies exist which might be implemented to reduce dietary salt intake within the population. An initial literature review suggested three contrasting policy options with evidence of effectiveness: 1) a nationwide health promotion campaign which would raise awareness and encourage people to reduce their salt intake, 2) requiring manufacturers to clearly label food products stating the salt content of the product to encourage people to opt for lower salt levels, 3) mandatory reformulation requiring manufacturers of food products to lower salt content. Given that these policies could also be implemented in combination as well as individually, six permutations were considered for evaluation (Table 1).

**Table 1:** Salt reduction policies for evaluation and estimated effectiveness of policy.

Policy	Effectiveness [Range]	Reference
Health promotion campaign	5% [1%–35%]	[31]
Labelling of food packaging (labelling)	10% [5%–15%]	[6]
Mandatory salt reduction of processed foods (reformulation)	10% [5%–40%]	[10]
Health promotion campaign in conjunction with labelling of food packaging	15% [10%–20%]	[11]
Health promotion campaign in conjunction mandatory salt reduction of processed foods	15% [15%–30%]	[11]
All three policies in combination	30% [10%–50%]	[32]

### Effectiveness of different policies

A literature review was conducted to obtain estimates of the effectiveness of each of the policies. In a step wise approach the review was narrowed to include only studies reporting estimates of the effectiveness of each of these policies, including evidence on the effectiveness of the combined policies, on changing the behaviour of the general population and not targeted at a specific group. Databases searched included Medline, Embase and Econlit (search strategy available in Supporting Information S1). Abstracts were checked for studies which included effectiveness estimates for any of the selected six policies. For each policy effectiveness estimates which were considered to be the most recent and reliable based on the outcomes of previous rigorous systematic reviews or observed changes in population salt intake from large trials were selected. To account for uncertainty around this value a minimum and maximum effectiveness estimate was also included as a range around this ‘best’ estimate. The final effectiveness values used in the analysis and the references at which each estimate was based on are presented in Table 1.

### Health Outcomes

We extended the four country IMPACT CHD models [17], [18] to quantify the effect of the predefined salt reduction policies on CHD mortality, and to estimate the resulting gain in life-years, based on the method described by Unal and Fidan [19], [20]. A detailed description of the basic IMPACT Model is available in Supporting Information S2.

First, we used the values resulting from our literature review as estimates of the expected reduction of current sodium salt consumption attributable to each given policy (Table 1). The expected change in salt intake was then translated into a change in mean population blood pressure based on the effects meta- analysis estimates provided by He and McGregor [3]. The resulting change in blood pressure was used to estimate the number of deaths prevented or postponed (DPPs) in ten years (as a predefined time frame), using the IMPACT CHD Policy model beta approach, (see table S2.1 in Supporting Information S2) [21]. This was compared with the number of CHD deaths that would have been expected if the CHD model death rates of the baseline year continued (i.e. the ‘do nothing scenario’).

We then calculated the number of life-years potentially gained by multiplying the estimated DPPs by the median survival for the different subgroups within the population (diagnosed CHD, undiagnosed CHD and population free of CHD). Estimates of median survival were obtained from a previous analysis performed for England and Wales for 1990–2000, based on large linked population based datasets, and community based cohorts [22], [23]. These were considered a reasonable historical proxy for current median survivals in the countries analysed given the lack of local longitudinal data [19], [20]. A detailed description of the data sources and quality is available in Supporting Information S2.

The reduction in numbers of CHD patients was calculated by estimating the effect of the policy on hypertension prevalence based on the shift of SBP and DBP distributions and assuming a constant proportion of uncontrolled hypertension patients, and estimating the change in attributable cases using a population attributable risk fraction approach, using INTERHEART odds ratio [24].

### Costs

The cost data were split into three categories; 1) costs to the public sector of introducing each policy, 2) costs to the private sector of labelling packaging and reformulating food products 3) the costs to the health services of treating people with CHD

The cost to the public sector associated with implementing the health promotion campaign included promotional materials (posters, leaflets, billboards) and publicity through television and radio advertisements. These estimates were based on the cost of previous health promotion campaigns within each country and considered both material and human resources. We also assumed that the labelling of packaging and the reformulation of food products would generate a cost to the public sector to develop and enforce a law to require producers to comply with the policy. These costs were obtained from official departments in each country using a pre-tested standardized questionnaire. For the costs to the private sector, the cost of labelling packaging with the salt content and the cost to reformulate products were obtained through an interview-based survey of selected manufacturers within each country. We interviewed local producers of main food items that were considered major sources of dietary salt such as; dairy products, bakery, butter and margarines, pickles and salty snacks. An estimate of the likely increase in production and marketing costs of the reformulated/repackaged products in each country was obtained. The total costs of implementing the health promotion campaign, labelling of packaging, reformulation and monitoring for each country are presented in Table 2.

**Table 2:** Total Cost of implementing Policy (Excluding Health Care Costs) in PPP$.

Policy	Tunisia	Syria	Palestine	Turkey
health promotion campaign	101,407	331,690	47,593	5,287,500
labelling of food packaging (labelling)	44,067	180,648	47,153	118,772,305
Monitoring – labelling	22,963	163,269	12,808	1,680,229
Mandatory salt reduction of processed foods (reformulation)	113,988	96,166,052	9,622,631	197,009,853
Monitoring - reformulation	22,963	163,269	12,808	1,680,229

Health care costs were considered using the IMPACT model. The main CHD conditions were identified, these were: acute myocardial infarction (AMI), secondary prevention after AMI, unstable angina, chronic angina, heart failure admitted into hospital and heart failure treated in the community) and hypertension. For each CHD event a treatment package was constructed including all drugs, procedures and associated medical professional time. The costs of each of these items were obtained from the Ministry of Health in each country based on typical reimbursement rates. The IMPACT model includes the frequency of use, uptake rates and patient numbers and from this the total cost of the CHD event per patient could be calculated (Table 3 details the total cost per condition for each country).

**Table 3:** Unit Cost per patient for CHD Event in PPP$.

CHD Event	Tunisia	Syria	Palestine	Turkey
Acute AMI	14,273	381	2,333	1,975
Secondary prevention following AMI	1,145	118	595	604
Unstable Angina	11,285	51	1,062	3001
Chronic Angina	2,201	897	623	434
Chronic Heart Failure – treatment In hospital	3,429	129	342	594
Chronic Heart Failure –treatment in the community	394	269	465	180
Hypertension	204	55	212	67

All costs were collected in 2010 prices in local currency. To allow for comparison between countries all costs were converted to international dollars using purchasing power parity (PPP) exchange rates.

### Cost Effectiveness Analysis

A ten year time horizon was taken for the analysis. The total cost of each policy option was calculated as the sum of the cost of introducing the policy and the total CHD events related health care costs over the 10 years. For the health promotion campaign, it was assumed that the campaign would be repeated each year. For the labeling and reformulation policies, it was assumed that there would be an initial set up cost in the first year, but in the subsequent years the only cost would be for monitoring to ensure compliance. All future costs and outcomes were discounted at 3% [25].

Each policy was compared against a baseline scenario of ‘doing nothing’. For this scenario, the current number of CHD patients was extracted from the IMPACT model and it was assumed that broadly similar numbers of CHD patients would occur over the 10 year time frame. The incremental cost and LYG of each policy over the baseline was calculated, and the incremental cost per LYG for each policy was then elicited.

### Sensitivity Analysis

Sensitivity analysis was conducted to assess the robustness of the results. The uncertainty surrounding the effectiveness of the policy to reduce population salt intake and its impact on total costs per LYG was calculated using the minimum and maximum effectiveness values extracted from the literature review (as shown in Table 1).

### Ethics Statement

An ethics statement was not required for this project.

## Results

All policies in all countries gained life years compared with the baseline scenario. Table 4 gives the total discounted costs saved (incremental costs), discounted cost per capita (incremental cost per capita) and life years gained for each policy in the four countries. A full set of results for each of the four countries, including undiscounted values, are presented in Supporting Information S3 (Tables S3.1–S3.4). In Tunisia, all policies were cost saving apart from health promotion. In Syria, policies involving health promotion and labelling were cost saving. However reformulation costs were high therefore only the policy which combined reformulation with health promotion and labelling was cost saving. Furthermore, the incremental cost per LYG of the policies which involved reformulation were all low (below $5000). Similarly in Palestine, all policies were cost saving apart from the reformulation using discounted costs. In Turkey all policies were cost saving.

**Table 4:** Discounted Costs and Effectiveness of Interventions over 10 year period.

	Tunisia			Syria			Palestine			Turkey		
Policy	Cost Saved* ($ PPP, millions)	Cost Saved* per Person ($PPP)	Life Years Gained	Cost Saved* ($ PPP, millions)	Cost Saved* per Person ($PPP)	Life Years Gained	Cost Saved* ($ PPP, millions)	Cost Saved* per Person ($PPP)	Life Years Gained	Cost Saved* ($ PPP, millions)	Cost Saved* per Person ($PPP)	Life Years Gained
HP	−17	−2	1,151	5	0.3	5,679	7	2	479	949	13	68,816
L	39	4	2,272	34	1.7	11,192	9	2	945	1043	14	135,221
R	39	4	2,272	−61	−3.0	11,192	−0.13	−0.03	945	965	13	135,221
R+L	92	9	3,361	−35	−1.7	16,543	−2	−0.4	1,398	992	14	199,303
R+HP	84	8	3,361	−36	−1.8	16,543	−2	−0.4	1,398	1079	15	199,303
3 Policies	235	22	6,455	39	1.9	31,674	6	2	2,682	1324	18	199,303

*negative values indicates the incremental cost of the policy compared with baseline.

For policy purposes it is also important to know how the costs incurred and costs saved are split between different sectors. In Tables 5, 6, 7, 8, the total costs for each policy are disaggregated into costs to the private sector, the non health care public/governmental sector and the health service. The cost savings arise through reductions in health service costs because of the reduced number of CHD events compared with the baseline.

**Table 5:** Tunisia - Disaggregated total cost.

Policy	Private Sector	Public Sector (non health care)	Health Care Costs	Total Cost	Discounted Private Sector	Discounted Public Sector (non health care)	Discounted Health Care Costs	Discounted Total Cost
**Baseline**	0	0	6,008,373,571	6,008,373,571	0	0	5,142,764,646	5,142,764,646
**Health Promotion**	0	1,014,074	5,987,515,951	5,988,530,025	0	867,979	5,159,603,287	5160,471,266
**Labelling**	44,067	229,630	5,920,865,316	5,921,139,012	44,067	196,548	5,103,196,609	5,103,437,223
**Reformulation**	113,988	263,778	5,920,865,316	5,921,243,082	113,988	225,776	5,103,196,609	5,103,536,373
**Reform +Labelling**	158,055	527,556	5,857,491,540	5,858,177,151	158,055	451,552	5,049,563,149	5,050,172,756
**Reform + Health Promotion**	133,988	1,277,852	5,857,491,540	5,858,883,380	113,988	1,093,755	5,057,013,039	5,058,220,783
**All 3 Combined**	158,055	1,541,630	5,688,039,969	5,689,739,653	158,055	1,319,532	4,906,155,661	4,907,633,247

**Table 6:** Syria - Disaggregated total cost.

Policy	Private Sector	Public Sector (non health care)	Health Care Costs	Total Cost	Discounted Private Sector	Discounted Public Sector (non health care)	Discounted Health Care Costs	Discounted Total Cost
**Baseline**	0	0	3,667,466,226	3,667,466,226	0	0	3,139,105,021	3,139,105,021
**Health Promotion**	0	3,316,901	3,632,918,062	3,636,234,963	0	2,839,044	3,130,919,539	3,133,758,584
**Labelling**	180,648	1,632,687	3,599,425,228	3,601,238,563	180,648	1,397,471	3,102,574,430	3,104,152,549
**Reformulation**	96,166,052	1,632,687	3,599,425,228	3,697,223,967	96,166,052	1,397,471	3,102,574,430	3,200,137,953
**Reform +Labelling**	96,346,700	3,165,374	3,566,976,959	3,666,589,032	96,346,700	2,794,941	3,075,113,340	3,174,254,981
**Reform + Health Promotion**	96,166,052	4,949,588	3,566,976,959	3,668,092,598	96,166,052	4,236,515	3,075,113,340	3,175,515,907
**All 3 Combined**	96,346,700	6,582,275	3,475,780,825	3,578,709,799	96,346,700	5,633,986	2,997,933,717	3,099,914,402

**Table 7:** Palestine - Disaggregated total cost.

Policy	Private Sector	Public Sector (non health care)	Health Care Costs	Total Cost	Discounted Private Sector	Discounted Public Sector (non health care)	Discounted Health Care Costs	Discounted Total Cost
**Baseline**	0	0	354,719,519	354,719,519	0	0	303,616,109	303,616,109
**Health Promotion**	0	475,933	343,755,934	344,231,866	0	407,367	296,373,821	296,781,187
**Labelling**	47,153	128,078	340,961,746	341,136,976	47,153	109,626	294,009,090	294,165,868
**Reformulation**	9,622,631	128,078	340,961,746	350,715,454	9,622,631	109,626	294,009,090	303,741,346
**Reform +Labelling**	9,669,784	256,155	338,456,478	348,382,416	9,669,784	219,252	291,888,872	301,777,907
**Reform + Health Promotion**	9,622,631	604,010	338,456,478	348,683,119	9,622,631	516,992	291,888,872	302,028,495
**All 3 Combined**	9,669,784	732,088	332,236,324	342,638,196	9,669,784	626,618	286,624,733	296,921,135

**Table 8:** Turkey - Disaggregated total cost.

Policy	Private Sector	Public Sector (non health care)	Health Care Costs	Total Cost	Discounted Private Sector	Discounted Public Sector (non health care)	Discounted Health Care Costs	Discounted Total Cost
**Baseline**	0	0	20,004,324,977	20,004,324,977	0	0	17,122,359,991	17,122,359,991
**Health Promotion**	0	52,875,000	18,878,025,956	18,932,900,956	0	45,257,452	16,284,001,991	16,329,259,444
**Labelling**	118,772,305	16,802,290	18,693,483,264	18,829,057,859	118,772,305	14,381,633	16,127,822,831	16,260,946,496
**Reformulation**	197,009,853	16,802,290	18,693,483,264	18,907,295,407	197,009,853	14,381,633	16,127,822,831	16,339,214,317
**Reform +Labelling**	315,782,158	33,604,580	18,289,265,837	18,638,652,575	315,782,158	28,763,266	15,785,732,134	16,130,277,558
**Reform + Health Promotion**	197,009,853	69,677,290	18,478,017,815	18,744,704,958	197,009,853	59,639,085	15,945,473,623	16,202,122,566
**All 3 Combined**	315,782,158	86,479,580	18,478,017,815	18,880,279,553	315,782,158	74,020,718	15,945,473,623	16,335,276,504

Sensitivity analysis was performed to calculate the total costs and LYG assuming different levels of effectiveness. Using the minimum and maximum effectiveness estimate for each policy (as outlined in Table 1), the incremental cost and life years gained for each policy in each country is presented in Tables 9, 10, 11, 12. For the maximum estimates, all policies in all countries were cost saving apart from the policy of reformulation combined with health promotion in Syria.

**Table 9:** Tunisia – Sensitivity Analysis.

Policy	Minimum Effectiveness estimates, Costs saved* Against Baseline ($PPP)	Minimum Effectiveness estimates, Life Years Gained	Maximum Effectiveness estimates, Costs saved Against Baseline ($PPP)	Maximum Effectiveness estimates, Life Years Gained
**Health Promotion**	−64,733,792	233	277,608,378	7,431
**Labelling**	−17,079,255	1,151	92,960,882	3,361
**Reformulation**	−17,178,405	1,151	317,064,401	8,380
**Reformulation + Labelling**	92,591,889	3,361	235,999,378	6,455
**Reformulation + Health Promotion**	30,910,403	2,272	135,299,982	4,421
**All 3 Policies together**	38,090,450	2,272	385,132,741	10,202

*negative values indicates the incremental cost of the policy compared with baseline.

**Table 10:** Syria – Sensitivity Analysis.

Policy	Minimum Effectiveness estimates, Costs saved* Against Baseline ($PPP)	Minimum Effectiveness estimates, Life Years Gained	Maximum Effectiveness estimates, Costs saved* Against Baseline ($PPP)	Maximum Effectiveness estimates, Life Years Gained
**Health Promotion**	−17,972,286	1,149	162,360,949	36,426
**Labelling**	6,607,363	5,679	62,413,562	16,543
**Reformulation**	−89,378,040	5,679	90,836,210	41,039
**Reformulation + Labelling**	−35,149,960	16,543	42,029,664	31,674
**Reformulation + Health Promotion**	−63,871,976	11,192	−9,824,086	21,737
**All 3 Policies together**	−65,450,094	11,192	130,384,153	49,866

*negative values indicates the incremental cost of the policy compared with baseline.

**Table 11:** Palestine – Sensitivity Analysis.

Policy	Minimum Effectiveness estimates, Costs saved* Against Baseline ($PPP)	Minimum Effectiveness estimates, Life Years Gained	Maximum Effectiveness estimates, Costs saved Against Baseline ($PPP)	Maximum Effectiveness estimates, Life Years Gained
**Health Promotion**	4,788,341	97	18,025,358	3,086
**Labelling**	7,085,509	479	11,570,459	1,398
**Reformulation**	−2,489,969	479	9,995,219	3,479
**Reformulation + Labelling**	1,838,202	1,398	7,102,340	2,682
**Reformulation + Health Promotion**	−532,604	945	87,146,361	1,838
**All 3 Policies together**	−689,383	945	11,627,340	4,232

*negative values indicates the incremental cost of the policy compared with baseline.

**Table 12:** Turkey.

Policy	Minimum Effectiveness estimates, Costs saved Against Baseline ($PPP)	Minimum Effectiveness estimates, Life Years Gained	Maximum Effectiveness estimates, Costs saved Against Baseline ($PPP)	Maximum Effectiveness estimates, Life Years Gained
**Health Promotion**	793,100,547	13,960	1,778,191,272	434,041
**Labelling**	861,383,222	68,816	1,203,473,919	199,303
**Reformulation**	783,145,674	68,816	1,714,028,326	487,712
**Reformulation + Labelling**	992,082,433	199,303	1,369,456,702	378,439
**Reformulation + Health Promotion**	920,237,425	135,221	1,214,365,624	261,147
**All 3 Policies together**	787,083,487	135,221	1,718,754,821	589,532

For the minimum effectiveness estimates more policies incurred an additional cost compared with baseline. In Tunisia, the labelling and reformulation policies had an incremental cost per LYG of $14,000 while for the health promotion policy the incremental cost per LYG rose to over $150,000. In Syria, the health promotion and labelling policies continued to be cost saving but all other policies resulted in additional costs. In Palestine, the three policies which involved reformulation were no longer cost saving using the minimum effectiveness estimates but were still cost effective. In Turkey, all policies were still cost saving.

## Discussion

Reducing dietary salt intake across the population appears an effective way of reducing coronary heart disease events and saving substantial costs in each of these four middle income countries.

Three contrasting policies in six permutations were evaluated. All of the policies resulted in a gain in life years compared with the baseline of no intervention. The majority of the policies were cost saving, with the biggest savings arising from a comprehensive approach which combined labelling and reformulation with a health promotion campaign. Even when a policy was not cost saving it would still be regarded as cost effective according to established cost effectiveness thresholds [25]. The cost savings which arise from each of these policies stem from a reduction in health care costs due to the potential reduction in the number of CHD events. In this study we assumed that governments would only be able to regulate local manufacturers of dairy, bread and snack food products and not multinational food producers importing food products into each of the four countries. This assumption seemed reasonable as the market share for locally produced dairy, bread and snack food products within each of these countries is high. Furthermore, most international food companies are continually reformulating their products to increase competitiveness and maximise profits in a changing environment. It is therefore likely that they are already reducing the salt content of their food because many Western countries already have voluntary policies to reduce salt in foods. If regulation was introduced it would simply steer this existing process more rapidly in a healthier direction, with minimal additional costs. The impact on the public sector overall is likely to be favourable as spending on the implementation of the policies would be offset against reduced spending on health care.

The impact of each policy in terms of both costs and outcomes is critically dependent on the assumed effectiveness of each policy intervention in reducing salt intake. In Table 4, the costs and outcomes reflect our ‘best’ estimate of effectiveness based on a detailed review of the literature. However, recognising the substantial uncertainties, a sensitivity analysis was conducted using widely separated minimum and maximum effectiveness estimates. For the maximum estimates, all policies in all countries were cost saving (apart from the policy of reformulation combined with health promotion in Syria), but these may overestimate the impact of these polices. The results of the minimum effectiveness estimates were more mixed. These represent a conservative account of what might be achieved, and yet still suggest substantial benefits and cost savings for many of the policies.

Our findings are consistent with those from high income countries which generally report large health gains across the population and cost savings, especially for policies involving reformulation of food products [6]. This study is the first to take the methodology previously applied in high income countries (which includes costs to both the public and private sectors as well as health care costs) and apply it to a middle income setting. It extends the work of Asaria et al [11] and Murray et al [12] for middle and low income countries which incorporated only the costs of setting up and running the intervention by the public sector and not the health care costs of treating CHD or costs to the private sector. Considering only the public sector costs in this study, the implementation of these six policy permutations would cost between PPP $ 0.02–0.13 per person in Tunisia, PPP$0.07–0.28 per person in Syria, PPP$0.03–0.15 per person in Palestine and PPP$0.20–1.02 per person in Turkey. Such information is reassuringly consistent with older studies, and might prove useful for policy makers in each country.

The analysis in this study is confined to a ten year time horizon. It does not take into account any health care costs postponed to the distant future [26]. Future analysis might include future unrelated health care costs, such as costs associated with productivity gains from changes in work force participation or changes in tax revenue following regulation, to give policy makers a more accurate picture of the total resource use of a policy. Furthermore, life time costs might also be lower [27].

The methodology for the collection of cost data varies across countries due to differences in availability of such data. This is a limitation of the study and therefore we need to interpret differences between the countries cautiously. However we did make major efforts to maximise compatibility, and the general findings appear robust. The effectiveness of each policy is based on the values observed in relatively few other countries. We used the most recent and robust estimates of effectiveness but also applied a robust sensitivity analysis to provide a better view of the potential minimum and maximum impact of these policies. The sensitivity analysis was limited to the effectiveness estimates due to data availability. Other model inputs such as the median survival were not varied in the analysis; if data become available for any of the specific countries this analysis could be repeated to improve the applicability of the results to the local situation. The sensitivity analysis we performed was not probabilistic due to the design of the IMPACT models that were available for the countries. The IMPACT model for each country was re-estimated using the maximum and then minimum effectiveness estimated and the total patient numbers for each CHD state and Life Years Gained was used to calculate the total incremental cost per LYG for each policy. The analysis assumes that the current (2010) level of CHD rates in each country will continue for the ten year time frame. Interestingly, age adjusted CHD mortality rates are rising in Tunisia and Syria, but falling in Palestine and Turkey. Our results may therefore underestimate the cost effectiveness of the policies in Tunisia and Syria and over-estimate those in Palestine and Turkey. Our cost effectiveness estimates may also be underestimated because the cost of reformulation was obtained from the manufacturers who may tend to exaggerate the true cost of reformulation.

We chose the interventions which appeared promising in terms of potential effectiveness. This was not a complete or comprehensive list; more a useful selection of three contrasting approaches.

We also simplistically assumed a single step change in policy. In reality implementation might be a phased over time as possibly being easier for industry and for consumers (less likely to detect progressive changes in salt content). Such phasing would slightly delay achieving the full benefits. We also assumed that the demand on the reformulated product will remain constant in the 10 years period.

These cost effectiveness data produced in this paper provide an important input into the decision making process. Implementation of a salt reduction policy in each of the four countries will be influenced by both the particular industrial environment as well as the preferences of policy makers. Policies which involve health promotion and labelling of food products may receive more support being perceived as easier to implement. In Turkey and Syria, manufacturers expressed concern about the reformulation policy as they believe reduced salt products might not be acceptable to consumers, mainly due to taste. In Palestine, manufacturers appeared to be more amenable to reformulation and policy makers preferred a one step programme of implementation as it was less costly, so that the combination of all 3 policies might therefore be the recommended strategy.

This paper also may be considered to be contributing evidence to the emerging field of nutrition economics which seeks to evaluate the health and economic outcomes of nutrition based interventions [28], [29]. The understanding of the role nutrition plays in public health will become increasingly important for policy makers when deciding on how best to allocate scarce health care resources especially the emphasis between preventative and curative care. At present more evidence is needed which links food consumption and the specific nutritional elements within that food to longer term health outcomes. Salt consumption is one area in which these links are already being made following longer term studies such as the North Karelia Project in Finland [30]. If these links can be established for other foods there is likely to be an increased need to develop the field of nutrition economics as has previously been done in health economics for curative treatments.

This study provides the first detailed evaluation of salt reduction policies in four middle income Eastern Mediterranean countries. The results powerfully reinforce the conclusions of previous studies in high income countries demonstrating the cost effectiveness of salt reduction policies. Decreasing dietary salt intake could generate substantial health benefits in terms of life years gained and cost savings.

## Supporting Information

Supporting Information S1 **Literature Search Strategy.**
(DOC)

Supporting Information S2 **IMPACT Model Methodology.**
(DOCX)

Supporting Information S3 **Full Results on Costs and Life Years Gained per Country.**
(DOCX)
