# Supplementary material for: A Cost Effectiveness Analysis of Salt Reduction Policies to Reduce Coronary Heart Disease in Four Eastern Mediterranean Countries
Source: PLoS One. 2014 Jan 7;9(1):e84445. doi: 10.1371/journal.pone.0084445 (PMC3883693; doi:10.1371/journal.pone.0084445)
Supplement: Supporting Information S1 — Literature Search Strategy. (DOC) [file pone.0084445.s001.doc]

Supporting Information 1: Literature Search Strategy

(Economic Evaluation OR Resource OR Incremental Cost Effectiveness Ration OR ICER) AND (Public Health Campaign OR Awareness Campaign OR Health Improvement Campaign OR Behaviour Change Campaign OR Food Legislation OR Food Labelling OR Product Labelling OR Content Labelling OR Nutrition Information OR Nutrition Labelling) AND (Heart Disease OR Hypertension OR Obesity OR Type 2 Diabetes) AND (Salt Reduction OR Salt Content OR Salt intake OR Salt Limit OR Salt Consumption)

Inclusion criteria

- All published literature available through Medline and EMBASE between January 1979 and May 2011
- Relevant grey literature from the same time period
- Published literature in English, or other languages where an English abstract is available online
- Articles with only English abstracts available through database searches
- Published literature relating to studies in any country
- Published literature relating to the three proposed interventions when implemented in general; and also when implemented in relation to food salt content
- Published literature relating to ‘intrinsic’ salt (i.e. already present in food at the point of purchase) and / or ‘extrinsic’ salt (i.e. salt added during cooking at home or at the table as food is served)

Exclusion criteria

- Published literature in another language than English, where an English abstract is unavailable online
