# Supplementary material for: A Cost Effectiveness Analysis of Salt Reduction Policies to Reduce Coronary Heart Disease in Four Eastern Mediterranean Countries
Source: PLoS One. 2014 Jan 7;9(1):e84445. doi: 10.1371/journal.pone.0084445 (PMC3883693; doi:10.1371/journal.pone.0084445)
Supplement: Supporting Information S2 — IMPACT Model Methodology. (DOCX) [file pone.0084445.s002.docx]

Supporting Information Section 2

**IMPACT Model methodology:**

IMPACT is a deterministic, cell-based policy model. It uses epidemiological information to estimate the contributions of population-level risk factor changes (impacting mainly on incidence) and changes in the uptake of evidence-based treatments (impacting mainly on case fatality) between two points in time (the start-year and the end-year). The primary outcome measure of the model is the deaths prevented or postponed, if mortality is declining (DPPs), or the increase in mortality, in populations where CHD mortality rates are rising. This model has been validated in Europe, New Zealand, China and the United States, and implemented on over 20 populations [[1](#_ENREF_1),[2](#_ENREF_2),[3](#_ENREF_3),[4](#_ENREF_4)].

The starting point for the model is to calculate the ‘target’ number of deaths the model needs to explain. This target number is obtained by using death counts recorded in the official registration system to calculate the difference between the actual observed Coronary Heart Disease (CHD) deaths recorded in the end-year from expected deaths, i.e. simple indirect standardisation: the number that would have occurred in the end-year had the CHD mortality rates remained the same as in the start-year.

The calculation of the modelled estimate of DPPs rests on utilising two well-studied relationships: firstly, that between risk factor change and the relative reduction in CHD mortality; secondly, that between treatment uptake and reductions in case-fatality in patients with a specific form of CHD.

The model applies the relative risk reduction quantified in previous randomised controlled trials and meta-analyses to estimate the mortality reduction attributable to:

a) temporal change in risk factor prevalence (in those without diagnosed CHD) to calculate the DPPs ‘explained’ by specific risk factor trends;

b) net change over the period in the uptake of specific treatments in patients with each specific form of CHD to estimate DPPs ‘explained’ owing to improved 1-year case fatality rates. Great care is taken to avoid double counting the same individuals.

The mortality benefits from the risk factor reduction in the population, and the treatment benefits in patient groups are then summed. This mortality sum represents the deaths prevented or postponed (DPPs) ‘explained’ by the model.

At the end of the modelling process, the total DPPs ‘explained’ by the model is then compared with the observed fall in deaths (the ‘target’ to be explained).

Model fit is therefore calculated as the difference between the observed deaths and model DPPs, and expressed as the percentage explained. This measures the extent to which the model was successful in explaining the observed change in CHD mortality in the population.

A policy model like IMPACT thus stands in contrast to a typical multivariate regression model. A typical multivariate regression model represents a statistical approach to describing a single data-set, for instance generated by a single cohort or RCT. In contrast, a policy model such as IMPACT seeks to integrate and synthesise best estimates from a variety of sources to reliably estimate the extent to which a range of factors, acting in combination, explain or predict an outcome. We did not obtain the parameters for this model by running regressions. Rather, the model incorporates the best coefficients from the largest meta-analysis or randomised controlled trials of the reduction in case fatality attributed to treatment or the independent effect sizes of a unit change in each risk factor on CHD mortality.

**Changes in mortality rates from Coronary Heart Diseases (CHD) between base year and final year of model**

- 1. **Expected and observed number of deaths from CHD, then calculating the DPPs:**

We first calculated the expected CHD number of deaths in the final year (generally around 2009) had base year (generally around 1997) age and sex specific mortality rates remained constant from the base year. This was calculated using indirect standardisation, by multiplying age and gender specific mortality rates in the base year by the population size for each 10-year age-gender stratum in the final year. The primary output of the IMPACT model is the number of **deaths prevented or postponed** (DPPs) if mortality is declining, or increase in deaths, if mortality is increasing, due to the changes in CHD mortality rates since the base year. This was then calculated as the difference between the number of CHD deaths expected in the final year, and the number of CHD deaths actually observed in the final year.

- 1. **Estimating DPP attributable to evidence based treatments:**

The number of DPPs attributable to treatments was estimated from information on the size of the clinically relevant patient groups, each intervention relative risk reduction reported in systematic reviews and meta-analyses; intervention uptakes, and case fatality rates in the absence of any treatment.

- 1. **Potential overlaps between patient groups:**

There are potential overlaps between patient groups (meaning that one person may belong to more than one patient group at the same time) . Hospital patient groups were selected based on one-year case fatality and overlapping between groups was avoided.

**Calculating treatment DPPs**

For each of the groups, we estimated the number of DPPs that were attributable to various treatments. The deaths prevented or postponed associated with a specific CHD treatment within a disease subgroup was estimated by taking the product of the number of people in the subgroup the proportion of those patients who received a particular treatment case fatality rates, DPPs for at least one year were considered in the calculation based on survival benefit over a one year time interval, and the relative risk reduction attributed to that specific treatment based on the published literature. We assumed that compliance defined as the proportion of patients prescribed medications on therapeutic doses of medication, was 100% among hospital patients, 70% among symptomatic community patients and 50% in asymptomatic individuals taking statins or anti-hypertensives for primary prevention. All these assumptions were tested in subsequent sensitivity analyses.

**EXAMPLE 1: estimation of DPPs from a specific treatment**

In Palestine in 2009, 112 men aged 55-64 were hospitalized with AMI. Utilization of aspirin was 49%. Efficacy of aspirin is 15%[[5](#_ENREF_5)] 1-year case-fatality rate was 7.9%.

The deaths prevented or postponed (DPPs) was calculated as:

***Patient numbers x treatment uptake x relative mortality reduction x one-year case fatality***

***=*** 112x 49% x 15% X 6.4% = 25 deaths prevented or postponed.

1. **Risk factors**

The IMPACT model calculates the DPPs associated with changes in CHD risk factors, including smoking, total cholesterol, systolic blood pressure, body mass index, diabetes mellitus, and physical inactivity.

Two approaches were used to calculate DPPs, the **regression approach** and the **population-attributable risk factor (PARF) approach;** The regression approach was used for continuous variables (systolic blood pressure, total cholesterol, and body mass index). The number of expected deaths from CHD occurring in 2005 (the end year) was multiplied by the absolute change in risk factor prevalence, and by a regression coefficient quantifying the change in CHD mortality that would result from the change in risk factor level. Natural logarithms were used, assuming a log-linear relationship between changes in risk factor levels and mortality.

**EXAMPLE 2: estimation of DPPs from risk factor change using the regression method:**

***Mortality fall due to reduction in systolic blood pressure in women aged 55-64***

In 2009, there were 20 CHD expected deaths (had 1998 mortality rates remained constant) among 43,693 women aged 55-64 years. Mean systolic blood pressure decreased by 3.4 mmHg (from 137.9 in 1998 to 134.5 mmHg in 2009). For every 20 mmHg reduction in systolic blood pressure, we estimated an age- and sex specific reduction in mortality of 50 percent. This generates a logarithmic coefficient of –0.035[[6](#_ENREF_6)].

The number of deaths prevented or postponed:

= (1-(EXP(coefficient*change))*expected deaths in 2009)

= (1-(EXP(-0.035*3.4))* 71)

= 8 DPPs

The PARF approach was used for categorical variables (smoking, diabetes, and physical inactivity). PARF was calculated as:

**(P x (RR-1)) / (P x (RR-1)) +1**

where P is the prevalence of the risk factor and RR is the relative risk for CHD mortality associated with that risk factor. DPPs were then estimated as the expected CHD deaths in 2009 multiplied by the difference in the PARF for 1998 and 2009.

**EXAMPLE 3: estimation of DPPs from risk factor change using the PARF method**

The prevalence of diabetes among men aged 65-74 years was 22% in 1999 and 28% in 2009. Assuming a Relative Risk of 1.93[[7](#_ENREF_7)], the PARF was 0.17 in 1998 and 0.21 in 2009. The number of deaths attributable to the increase in diabetes prevalence from 1998 to 2009 was therefore:

(144) * ( 0.21-0.17 ) = 6 DPPs

1. **Other Methodological Considerations**
   1. **Systolic BP and Hyperlipidemia**

In order to separate the DPPs from pharmacological versus non-pharmacological primary prevention of hypertension and hyperlipidemia, we subtracted the age-gender specific DPP’s calculated in the treatment section (i.e. for primary hyperlipidemia and hypertension patient groups), from the DPPs calculated in the risk factor section.

- 1. **Polypharmacy Issues**

There is a paucity of data on the efficacy of treatment combinations. Simply assuming that the efficacy of multiple treatments was additive would over-estimate the treatment effect; we therefore we used the Mant and Hicks method to estimate case-fatality reduction by polypharmacy for all treatments evaluated[[8](#_ENREF_8)].This approach was subsequently endorsed by Yusuf[[9](#_ENREF_9)]and Law and Wald [[10](#_ENREF_10)]. This approach estimates a cumulative relative benefit as follows:

Relative Benefit = 1 - ((1-relative reduction in case-fatality rate for treatment A) X (1- relative reduction in case-fatality rate for treatment B) X ...X (1- relative reduction in case-fatality rate for treatment N).

**EXAMPLE 4: estimation of reduced benefit if patient taking multiple medications (Mant and Hicks approach)**

For AMI survivors, applying relative risk reductions (RRR) for aspirin, beta-blockers ACE inhibitors statins and rehabilitation then gives:

***Relative Benefit = 1 - [(1 –aspirin RRR) X (1 - beta-blockers RRR) X (1 - ACE inhibitors RRR) X (1- statins RRR) X (1- rehabilitation RRR)]***

**= 1 - [(1- 0.15) X (1-0.23) X (1-0.20) X (1- 0.22) X (1- 0.26)]**

**= 1 - [(0.85) X (0.77) X (0.80) X (0.78) X (0.74)]**

**= 0.70 i.e. a 70% lower case fatality**

**c. Sensitivity Analyses**

Because of the uncertainty surrounding many of the values, multi-way sensitivity analyses were performed[[11](#_ENREF_11)]. For each model parameter, a maximum and minimum plausible value was assigned using the 95% confidence intervals from the source documentation; if this was unavailable, we defined these limits as 20% above and below the best estimate. The maximum and minimum plausible values were fed in to the model generating maximum and minimum estimates for deaths prevented or postponed.

**Summary of data sources for each country used in the baseline IMPACT models.**

| **Table S2.1 Data sources, data variations and assumptions made in the four countries** | | | | |
| --- | --- | --- | --- | --- |
| **Data** | **Tunisia** | **Syria** | **oPt** | **Turkey** |
| ***Study Years*** | **1997 – 2009** | **1996 – 2006** | **1998 – 2009** | **1995 – 2008** |
| ***Population*** | Tunisian National Institute of Statistics for 1997 and 2009 | Syrian bureau of Statistics. | the Palestinian Central Bureau of Statistics (PCBS) (2009) | Census and Address Based Population Registration System (2008) |
| ***Number of CHD deaths*** | National Public Health Institute - Tunisian Ministry of Public Health | WHO Global Health Observatory [[12](#_ENREF_12)]. | the Health Information Management Centre -Palestinian Ministry of Health | Turkish Statistical Institute |
| ***CHD hospital admissions*** | Tunisian Epidemiological Study “TEPS-ACS 2009”[[13](#_ENREF_13)] | Uptake survey conducted by SCTS specifically for this project in 2009. | Uptake survey conducted in 2009 | TUIK Hospital admission stats [[14](#_ENREF_14),[15](#_ENREF_15)], and the Framingham risk score |
| ***Secondary prevention*** | Premise I and Premise II[[16](#_ENREF_16)] [[17](#_ENREF_17)]. |  |  |  |
| ***Treatment uptake levels early treatment*** | Tunisian Epidemiological Study “TEPS-ACS 2009[[13](#_ENREF_13)]” | Treatment uptake survey conducted by SCTS. | National health surveys and treatment uptake surveys | Hospital survey and several studies published on treatment uptakes in the emergency department [[5](#_ENREF_5)] |
| ***treatment uptake in the community*** | Survey conducted by the Cardiovascular Epidemiology and Prevention Research Laboratory. | Outpatient survey conducted by SCTS in 2009 | Expert opinion from practising clinicians. | Information was based on (PREMISE [[18](#_ENREF_18)], EUROASPIRE III [[19](#_ENREF_19)]. |
| ***Risk factor trends*** | Cross-sectional surveys from representative sample from seven administrative regions | STEPwise survey by WHO in 2003. The Aleppo Diabetes Survey (ADS) conducted in 2006 [[20](#_ENREF_20)]. extrapolated data from the Palestinian authority for 1996 | Published epidemiological studies for that period.[[21](#_ENREF_21)] [[22](#_ENREF_22)]. | Base year: national surveys TEKHARF [[23](#_ENREF_23)], TURDEP [[24](#_ENREF_24)] supplemented with regional studies.  later years more national surveys[[25](#_ENREF_25)] [[26](#_ENREF_26)] |
| ***Potential overlaps*** | Potential overlaps between different groups of patients were identified and appropriate adjustments were made. | | | |
| ***Adjustments for total number of deaths, if any*** |  |  |  | Inflating the urban death numbers proportional to rural population assuming same mortality pattern exist in rural area. Total number of deaths was inflated also by 12% in men and 16% in women |

**Identification and assessment of relevant data**

**Tunisia**

Validated information on the Tunisian **population demographic changes** was obtained from Tunisian National Institute of Statistics for 1997 and 2009)[[27](#_ENREF_27),[28](#_ENREF_28)]. **Numbers of deaths for both years** were obtained from the National Public Health Institute - Tunisian Ministry of Public Health [[28](#_ENREF_28),[29](#_ENREF_29)]. **Population risk factor trend data** for 1997 and 2009 were obtained from national and regional epidemiological studies conducted in the community. The surveys were cross-sectional and the target population based on a nationally representative, stratified cluster sample of households according to the seven administrative regions of Tunisia and included both urban (65.9%) and rural areas (34.1% )[[30](#_ENREF_30),[31](#_ENREF_31),[32](#_ENREF_32),[33](#_ENREF_33),[34](#_ENREF_34)].

**The numbers of hospital admissions with CHD in addition to treatment uptake** were obtained from the Tunisian Epidemiological Study “TEPS-ACS 2009” conducted in 2009 in the MedCHAMPS Project which included 5 of the most important Tunisian hospitals [[13](#_ENREF_13)][[REF]](#_ENREF_58) .

Data concerning secondary prevention was obtained from Premise I (Prevention of recurrence of Myocardial Infarction and Stroke) conducted by the Cardiovascular Epidemiology and Prevention Research Laboratory in 2002 [[16](#_ENREF_16)][[REF]](#_ENREF_63) and Premise II conducted in 2009 during the MedCHAMPS Project [[17](#_ENREF_17)].

The number of patients undergoing Coronary Artery Bypass Grafting (CABG) and Angioplasty were obtained from the National Health Insurance Fund. The prevalence of Angina, Heart Attack survivors and Congestive Heart Failure in the community were estimated based on national health surveys and treatment uptake survey conducted by the Cardiovascular Epidemiology and Prevention Research Laboratory. We have access to the raw data for all these studies. Data on treatment uptake in the community was supported by systematically eliciting expert opinion.

**Efficacy of therapeutic interventions:** used recent meta-analyses and large randomised controlled trials. The Mant and Hicks approach was used to correct for polypharmacy[[35](#_ENREF_35)].

#### *The change in coronary heart disease deaths*

First, the number of CHD deaths **expected** in 2009 was calculated by indirect age standardization assuming that 1997 mortality rates had persisted unchanged until 2009. The number of CHD deaths actually **observed** in 2009 was then subtracted. The difference between the two represents the rise or fall in coronary heart disease deaths (the number of deaths prevented or postponed) that the IMPACT model needed to explain.

Case-fatality data came from large, hospital-based studies conducted in TAHINA Project [[17](#_ENREF_17)]. Survival benefit over a one-year time interval was used for all treatments.

**Syria**

Syrian data on risk factors and current uptake of evidence-based treatments for CHD were identified through extensive search of published and unpublished data and complemented with specifically designed surveys.

Data used to populate the model included: a) population and CHD related mortality, b) patient numbers in specific CHD groups (Myocardial Infarction (MI), Congestive Heart Failure (CHF), Chronic Angina Pectoris (AP), c) uptake of specific medical and surgical treatments for CHD, and d) population trends in major cardiovascular risk factors. The main outcome of the model was the number of deaths prevented or postponed (DPP) due to the changes in risk factors or treatment uptake.

Information on the population demographic changes for Syria between 1996-2006 were obtained from the Syrian bureau of Statistics. Numbers were comparable with the numbers provided by the U.N. department of economics and social affairs [[36](#_ENREF_36)]. Numbers of CHD related deaths for both years were obtained from the WHO Global Health Observatory [[37](#_ENREF_37)]. These numbers were cross-validated with the data provided by the Aleppo Household Survey (AHS), in which mortality estimates were calculated on the basis of participant-reported deaths occurring in the past 5 years among their adult (>20 years) household members [[38](#_ENREF_38)]. Mortality data for 1996 were estimated based on 2006 and 1985 data obtained from the WHO Statistical Information System [[37](#_ENREF_37)].

Population risk factors trend data for the year 2006 were obtained from two epidemiological studies. The first one is the STEPwise survey conducted by WHO in the rural and urban areas of Syria in 2003 in which a nationally representative sample of 9184 participants were surveyed. The second one is the Aleppo Diabetes Survey (ADS) conducted in 2006 in which a representative sample of 1168 from the city of Aleppo (2^nd^ largest city with population ≈ 2.5 million) were surveyed [[20](#_ENREF_20)]. Syrian data for the year 1996 were not available; we therefore extrapolated data from the Palestinian authority, because these data were the most complete and standardized ones among neighbouring countries and because of the cultural similarities of both populations.

Data on CHD hospital admissions and treatment uptake for 2008 were obtained from a treatment uptake survey conducted by SCTS specifically for this project in 2009 and covered 6 major hospitals. Three of these hospitals provide cardiac care in the Aleppo province, and the other 3 were general hospitals.

Community treatment uptake data for 2008 were obtained from an outpatient survey conducted by SCTS in 2009 using a random sample of five health centres. Information on treatment uptake in the community was verified with the help of experts’ opinion using a special questionnaire about treatment of CHD.

**Occupied Palestine Territories**

Information on the West Bank population demographic changes was obtained and validated for the first year from 1997 census based projections and 2007 census based projections for the final year by the Palestinian Central Bureau of Statistics (PCBS) [[39](#_ENREF_39)]. Numbers of deaths for both years were obtained from the Health Information Management Centre -Palestinian Ministry of Health. Population risk factors trend data for the year 1998 was obtained from two epidemiological studies conducted in the rural and urban areas of Ramallah governorate in the West Bank. These were the only available published epidemiological studies in the West Bank for that period. They covered a rural and an urban site that were prototypic of many West Bank villages and urban sites [[21](#_ENREF_21),[22](#_ENREF_22)].

CHD numbers of hospital admissions in addition to treatment uptake were obtained from our Treatment uptake survey conducted in 2009 which included four hospitals in the north, centre and south of the West Bank. The number of patients undergoing Coronary Artery Bypass Grafting (CABG) and angioplasty were obtained from records in the two hospitals providing this service in the West Bank. The prevalence of angina, heart attack survivors and congestive heart failure in the community were each estimated on the basis of national health surveys and treatment uptake surveys. Information on treatment uptake in the community was also checked by eliciting expert opinion

**Turkey**

Data on causes of death were collected only from urban settings which comprised approximately 50%-68% of the total population over the years 1988-2008 [[40](#_ENREF_40)]. Therefore total number of deaths was estimated by inflating the urban death numbers proportional to rural population simply assuming same mortality pattern exist in rural area. Total number of deaths was inflated also by 12% in men and 16% in women to account underreporting of the deaths based on expert opinion[[41](#_ENREF_41)]. The number of CHD deaths by age and sex groups for 1995 and 2008 were obtained from the Turkish Statistical Institute coded according to International Classification of Diseases, 8^th^*Revision* (corresponds to ICD9 codes 410-414) [[42](#_ENREF_42)]. Coding inaccuracy is an important limitation of the mortality data in Turkey [[43](#_ENREF_43)]. Ill-defined codes and “other heart diseases” code included large number of deaths that were accounted approximately 30 to 40% of the total deaths during the study period [[43](#_ENREF_43)]. Therefore 50% of the “other heart disease” were allocated to CHD deaths through the years [[41](#_ENREF_41)].

CHD numbers of hospital admissions were estimated using best available sources. In Turkey**,** this was the Ministry of Health (MoH) hospital admission data and from two cohort studies [[5](#_ENREF_5),[44](#_ENREF_44),[45](#_ENREF_45)]. The number of patients undergoing Coronary Artery Bypass Grafting (CABG) and angioplasty were obtained from the MoH hospital admission data supplemented with information by experts from Turkish Cardiology Association and Cardiovascular Surgery Association. Treatment uptake levels for initial CHD treatments in the hospital were obtained from the hospital survey designed by the research team in the Dokuz Eylul University Hospital and several studies that published on treatment uptakes in the emergency department [[5](#_ENREF_5)].

The prevalence of angina, MI survivors and patients with congestive heart failure in the community were estimated based on representative population surveys in each country. In Turkey these included National Household Survey and Health Survey 2008. Information on treatment uptake in the community dwelling patients was based on most recent sources. In Turkey ,PREMISE [[18](#_ENREF_18)] EUROASPIRE III [[19](#_ENREF_19)] .

**The IMPACT model Beta Approach**

The IMPACT model beta approach to estimate deaths prevented or postponed attributable to changes in Systolic blood pressure

In the regression approach used for systolic blood pressure (SBP) in these analyses, the number of CHD deaths in the start year for each model after adjusting for population change over the model time period, were multiplied by the absolute change in risk factor level, and by a regression coefficient (‘beta’) in the natural logarithmic scale, quantifying the estimated relative change in CHD mortality that would result from a one-unit change in risk factor level (Table I).

B Coefficients for estimating the effect of a given change in SBP levels on CHD mortality

**Estimated β coefficients from multiple regression analyses for the relationship between absolute changes in population mean risk factors and percentage changes in coronary heart disease mortality for men and women, stratified by age. Data sources, values and comments.**

| **Systolic blood pressure** | | **Age group (years)** | | | | |
| --- | --- | --- | --- | --- | --- | --- |
|  | | **25-44** | **45-54** | **55-64** | **65-74** | **75+** |
|  | |  |  |  |  |  |
| **Men** (hazard ratio per 20 mmHg) | | 0.49 | 0.49 | 0.52 | 0.58 | 0.65 |
| Men (log hazard ratio per 1 mmHg) | | **-0.036** | **-0.035** | **-0.032** | **-0.027** | **-0.021** |
|  | |  |  |  |  |  |
| *Minimum* | | *-0.029* | *-0.028* | *-0.026* | *-0.022* | *-0.017* |
| *Maximum* | | *-0.043* | *-0.042* | *-0.039* | *-0.032* | *-0.025* |
|  | |  |  |  |  |  |
|  | |  |  |  |  |  |
| **Women** (hazard ratio per 20 mmHg) | | 0.40 | 0.40 | 0.49 | 0.52 | 0.59 |
| Women (log hazard ratio per 1 mmHg) | | **-0.046** | **-0.046** | **-0.035** | **-0.032** | **-0.026** |
| *Minimum* | | *-0.037* | *-0.037* | *-0.028* | *-0.026* | *-0.021* |
| *Maximum* | | *-0.055* | *-0.055* | *-0.042* | *-0.039* | *-0.031* |
|  | |  |  |  |  |  |
|  | |  |  |  |  |  |
| Source: Prospective studies collaborative meta-analysis ^18^ | | | | | | |
| Units: Percentage change in CHD mortality per 20 mmHg change in systolic blood pressure | | | | | | |
| **Strengths:** | Large dataset, includes US data, adjusted for regression dilution bias, consistent with randomised controlled trials, results stratified by age and sex, with 95% confidence intervals | | | | | |
| **Limitations:** | Some publication bias still possible | | | | | |

1. Bennett K, Kabir Z, Unal B, Shelly A, Critchley J, et al. (2006) Explaining the recent decrease in coronary heart disease mortality rates in irelan, 1985-2000. Journal of Epidemiology and Community Health 60: 322-327.

2. Critchley J, Liu J, Zhao D, Wei W, S C (2004) Explaining the increase in coronary heart disease mortality in Beijing between 1984 and 1999. Circulation 110: 1236-1244.

3. Ford ES, Ajani U, Croft J, Critchley J, D L, et al. (2007) Explaining the decrease in U.S deaths from coronary heart disease, 1980-2000. New England Journal of Medicine 356: 2388-2398.

4. Unal B, Critchley J, S C (2004) Explaining the decline in coronary heart disease mortality in England and Wales, 1981-2000. Circulation 109: 1101-1107.

5. Aslan BU, Karcioglu O, Aslan O, Ayrik C, Kulac E, et al. (2002) [Does the short-term mortality differ between men and women with first acute myocardial infarction?]. Anadolu Kardiyol Derg 2: 284-290.

6. Lewington S, Clarke R, Qizilbash N, Peto R, Collins R (2002) Prospective Studies Collaboration. Age-specific relevance of usual blood pressure to vascular mortality: a meta-analysis of individual data for one million adults in 61 prospective studies. Lancet 360: 1903-1913.

7. Yusuf S, Hawken S, Ounpuu S, Dans T, Avezum A, et al. (2004) Effect of potentially modifiable risk factors associated with myocardial infarction in 52 countries (the INTERHEART study): case-control study. Lancet 364: 937-952.

8. Mant J, Hicks N (1995) Detecting differences in quality of care: the sensitivity of measures of process and outcome in treating acute myocardial infarction. Bmj 311: 793-796.

9. Yusuf S, Pais P, Afzal R, Xavier D, Teo K, et al. (2009) Effects of a polypill (Polycap) on risk factors in middle-aged individuals without cardiovascular disease (TIPS): a phase II, double-blind, randomised trial. . Lancet 373: 1341-1351.

10. Wald NJ, M. L (2003) A strategy to reduce cardiovascular disease by more than 80%. . BMJ 326: 1419.

11. Briggs A, Sculpher M, M. B (1994) Uncertainty in the economic evaluation of health care technologies: the role of sensitivity analysis. Health economics 3: 95-104.

12. Abdullah AS, CG.. H ( 2004) Promotion of smoking cessation in developing countries: a framework for urgent public health interventions. Thorax 59: 623-630.

13. Cardiovascular Epidemiology and Prevention Research Laboratory ( 2009) Tunisian Epidemiological Study on Acute Coronary Syndrome “TEPS-ACS 2009” MedCHAMPS Project.

14. Aslan BU, Karcioglu O, Aslan O, Ayrik C, Kulac E, et al. (2002) [Does the short-term mortality differ between men and women with first acute myocardial infarction?]. AnadoluKardiyolDerg 2: 284-290.

15. Badillioglu OUBUR (2011) Five-year incidence of coronary heart disease and risk factors in Güzelbahçe, İzmir. Turkish Journal of Public Health 9.

16. Ben Mansour N, Lassoued O, Saidi O, Aissi W, Ben Ali S, et al. (2012) Trends in Secondary Prevention of Coronary Heart Disease in Tunisia Prevention of Recurrences of MI and Stroke, 2002–2009. . Global Heart 7: 361-366.

17. Cardiovascular Epidemiology and Prevention Research Laboratory (2006) TAHINA project Final Report. Epidemiological Transition and Health Impact in North Africa.

18. Mendis S, Abegunde D, Yusuf S, Ebrahim S, Shaper G, et al. (2005) WHO study on Prevention of REcurrences of Myocardial Infarction and StrokE (WHO-PREMISE). . Bull World Health Organ 83: 820-829.

19. Tokgözoğlu L KE, Erol C, O; E, EUROASPIRE III Turkey Study Group (2010) [EUROASPIRE III: a comparison between Turkey and Europe]. Turk Kardiyol Dern Ars 38: 164-172.

20. Al Ali R, Rastam S, Fouad FM, Mzayek F, Maziak W (2011) Modifiable cardiovascular risk factors among adults in Aleppo Syria. Int J Public Health.

21. Abdul-Rahim HF, Abu-Rmeileh NM, Husseini A, Holmboe-Ottesen G, Jervell J, et al. (2001) Obesity and selected co-morbidities in an urban Palestinian population. Int J Obes Relat Metab Disord 25: 1736-1740.

22. Husseini A (2002) Type 2 Diabetes Mellitus and selected associated factors in an adult Palestinian population: an epidemiologic study of type 2 Diabetes Mellitus and impaired glucose tolerance (IGT) in Kobar and Ramallah, Palestine Oslo: University of Oslo.

23. Onat A, Senocak MS, Surdum-Avci G, ; OE (1993) Prevalence of coronary heart disease in Turkish adults. IntJCardiol 39: 23-31.

24. Satman I, Yilmaz T, Sengul A, Salman S, Salman F, et al. (2002) Population-based study of diabetes and risk characteristics in Turkey: results of the turkish diabetes epidemiology study (TURDEP). . Diabetes Care 25: 1551-1556.

25. Erem C, Hacihasanoglu A, Deger O, Kocak M, M. T (2008) Prevalence of dyslipidemia and associated risk factors among Turkish adults: Trabzon lipid study. Endocrine 34: 36-51.

26. Sanisoglu SY, Oktenli C, Hasimi A, Yokusoglu M, ; UM (2006) Prevalence of metabolic syndrome-related disorders in a large adult population in Turkey. BMC Public Health 6:.

27. National Institute of Statistics Tunisia (1997) Yearly Statistics Report (No. 40).

28. National Institute of Statistics Tunisia (2009) Yearly Statistics Report: N° 52.

29. National Public Health Institute Tunisia (2001 NPHI 2003) Deaths Registry,.

30. Ben Romdhane H (2001) Coronary Heart Diseases: the epidémic and its determinants. In: Institut NPH, editor. vol I: the risk factors Tunis.

31. Ben Romdhane HAHUP, 2009. Pour une urbanisation saine de la ville de l’Ariana. Enquête auprès des ménages. Octobre 2010 (2010) Ariana Healthy Urbanization Project, 2009. Pour une urbanisation saine de la ville de l’Ariana. Enquête auprès des ménages. Octobre 2010.

32. Institut national de nutrition et de technologie alimentaire Evolution de l’état nutritionnel de la population tunisienne. Enquête nationale 1996-1997.

33. Institut National de Santé Publique Institut National de Santé Publique. Epidémiologie des broncho-pneumopathies chroniques chez l’adulte. Résultats de l’enquête nationale menée en 1996-1997.

34. TAHINA project Final Report (2006) Epidemiological Transition and Health Impact in North Africa Cardiovascular Epidemiology and Prevention Research Laboratory,.

35. Mant J, N. H (1995) Detecting differences in quality of care: the sensitivity of measures of process and outcome in treating acute myocardial infarction. BMJ 311: 793-796.

36. United Nations (2011) Department of economics and social affairs.

37. World Health Organization (2004) WHO Global Health Observatory.

38. Maziak W, Ward KD, Mzayek F, Rastam S, Bachir ME, et al. (2005) Mapping the health and environmental situation in informal zones in Aleppo, Syria: report from the Aleppo household survey. Int Arch Occup Environ Health 78: 547-558.

39. Palestinian Central Bureau of Statistics (2007) Population projections in the Palestinian Territory, mid 2006. . Ramallah, Palestine: Palestinian Central Bureau of Statistics.

40. Turkish Statistical Institute (2012) Mortality statistics 1988-2008. Ankara.

41. Akgun S, Rao C, Yardim N, Basara B, Aydin O, et al. (2007) Estimating mortality and causes of death in Turkey: methods, results and policy implications. EurJPublic Health 17:. 593-599.

42. Turkish Statistical Institute (Turkstat) ( 2012) Demographic statistics,. Ankara.

43. Razum O, Akgun S, S. T (2000;) Cardiovascular mortality patterns in Turkey: what is the evidence? SozPraventivmed 45: 46-51.

44. Badillioglu O (2011) Five-year incidence of coronary heart disease and risk factors in Güzelbahçe, İzmir. Turkish Journal of Public Health 9.

45. Wilson PW, D'Agostino RB, Levy D, Belanger AM, Silbershatz H, et al. (1998) Prediction of coronary heart disease using risk factor categories. . Circulation 97: 1837-1847.
