# Supplementary material for: A Cost Effectiveness Analysis of Salt Reduction Policies to Reduce Coronary Heart Disease in Four Eastern Mediterranean Countries
Source: PLoS One. 2014 Jan 7;9(1):e84445. doi: 10.1371/journal.pone.0084445 (PMC3883693; doi:10.1371/journal.pone.0084445)
Supplement: Supporting Information S3 — Full Results on Costs and Life Years Gained per Country. (DOCX) [file pone.0084445.s003.docx]

Supporting Information 3

**Table S3.1 Tunisia - Total Cost and LYG**

| **Policy** | **Total Cost** | **Discounted Total Cost** | **Cost Saving against Baseline** | **Discounted Cost Saving against Baseline** | **Incremental Cost against Baseline** | **discounted Incremental Cost** | **Life-years gained (LYG) over Baseline** | **Incremental Cost per LYG** | **Incremental Cost per LYG (discounted)** |
| --- | --- | --- | --- | --- | --- | --- | --- | --- | --- |
| **Baseline** | 6,008,373,571 | 5,142,764,646 |  |  |  |  |  |  |  |
| **Health Promotion** | 5,988,530,025 | 5160,471,266 | 19,843,547 |  |  | 17,706,620 | 1151 |  | 15,377 |
| **Labelling** | 5,921,139,012 | 5,103,437,223 | 87,234,559 | 39,327,423 |  |  | 2272 |  |  |
| **Reformulation** | 5,921,243,082 | 5,103,536,373 | 87,130,490 | 39,228,273 |  |  | 2272 |  |  |
| **Reform +Labelling** | 5,858,177,151 | 5,050,172,756 | 150,196,421 | 92,951,889 |  |  | 3361 |  |  |
| **Reform + Health Promotion** | 5,858,883,380 | 5,058,220,783 | 149,490,190 | 84,543,863 |  |  | 3361 |  |  |
| **All 3 Combined** | 5,689,739,653 | 4,907,633,247 | 318,633,918 | 234,133,39 |  |  | 6455 |  |  |

**Table S3.2. Syria – Total Cost and LYG**

| **Policy** | **Total Cost** | **Discounted Total Cost** | **Cost Saving against Baseline** | **Discounted Cost Saving against Baseline** | **Incremental Cost against Baseline** | **discounted Incremental Cost** | **Life-years gained (LYG) over Baseline** | **Incremental Cost per LYG** | **Incremental Cost per LYG (discounted)** |
| --- | --- | --- | --- | --- | --- | --- | --- | --- | --- |
| **Baseline** | 3,667,466,226 | 3,139,105,021 |  |  |  |  |  |  |  |
| **Health Promotion** | 3,636,234,963 | 3,133,758,584 | 31,231,263 | 5,346,438 |  |  | 5,679 |  |  |
| **Labelling** | 3,601,238,563 | 3,104,152,549 | 66,227,663 | 34,952,472 |  |  | 11,192 |  |  |
| **Reformulation** | 3,697,223,967 | 3,200,137,953 |  |  | 29,757,740 | 61,032,931 | 11,192 | 2,659 | 5,453 |
| **Reform +Labelling** | 3,666,589,032 | 3,174,254,981 | 877,194 |  |  | 35,149,960 | 16,543 |  | 2,125 |
| **Reform + Health Promotion** | 3,668,092,598 | 3,175,515,907 |  |  | 626,372 | 36,410,886 | 16,543 | 38 | 2,201 |
| **All 3 Combined** | 3,578,709,799 | 3,099,914,402 | 88,756,427 | 39,190,619 |  |  | 31,67 |  |  |

**Table S3.3 Palestine– Total Cost and LYG**

| Policy | Total Cost | Discounted Total Cost | Cost Saving against Baseline | Discounted Cost Saving against Baseline | Incremental Cost against Baseline | discounted Incremental Cost | LYG over Baseline | Incremental Cost per LYG | Incremental Cost per LYG (discounted) |
| --- | --- | --- | --- | --- | --- | --- | --- | --- | --- |
| Baseline | 354,719,519 | 303,616,109 |  |  |  |  |  |  |  |
| Health Promotion | 344,231,866 | 296,781,187 | 10,487,653 | 6,834,921 |  |  | 479 |  |  |
| Labelling | 341,136,976 | 294,165,868 | 13,582,543 | 9,450,241 |  |  | 945 |  |  |
| Reformulation | 350,715,454 | 303,741,346 | 4,007,065 |  |  | 125,238 | 945 |  | 132 |
| Reform +Labelling | 348,382,416 | 301,777,907 | 6,337,103 | 1,838,202 |  |  | 1398 |  |  |
| Reform + Health Promotion | 348,683,119 | 302,028,495 | 6,036,400 | 1,587,614 |  |  | 1398 |  |  |
| All 3 Combined | 342,638,196 | 296,921,135 | 12,081,323 | 6,694,974 |  |  | 2682 |  |  |

**Table S3.4 Turkey – Total Cost and LYG**

| Policy | Total Cost | Discounted Total Cost | Cost Saving against Baseline | Discounted Cost Saving against Baseline | Incremental Cost against Baseline | discounted Incremental Cost | LYG over Baseline | Incremental Cost per LYG | Incremental Cost per LYG (discounted) |
| --- | --- | --- | --- | --- | --- | --- | --- | --- | --- |
| Baseline | 20,004,324,977 | 17,122,359,991 |  |  |  |  |  |  |  |
| Health Promotion | 18,746,358,264 | 16,173,080,284 | 1,257,966,713 | 949,279,707 |  |  | 68,816 |  |  |
| Labelling | 18,613,592,410 | 16,078,627,566 | 1,390,732,567 | 1,043,732,425 |  |  | 135,221 |  |  |
| Reformulation | 18,691,829,958 | 16,156,865,114 | 1,312,495,019 | 965,494,877 |  |  | 135,221 |  |  |
| Reform +Labelling | 18,638,652,575 | 16,130,277,558 | 1,365,672,402 | 992,082,433 |  |  | 199,303 |  |  |
| Reform + Health Promotion | 18,555,952,980 | 16,042,381,072 | 1,448,371,997 | 1,079,978,919 |  |  | 199,303 |  |  |
| All 3 Combined | 18,245,618,763 | 15,798,160,741 | 1,758,706,214 | 1,324,199,250 |  |  | 378,439 |  |  |
